# Perception versus Policy: Which Is More Important to Animal Welfare Volunteer Satisfaction?

**DOI:** 10.3390/ani14010095

**Published:** 2023-12-27

**Authors:** Laura Ann Reese

**Affiliations:** School of Planning, Design and Construction, Michigan State University, East Lansing, MI 48824, USA; reesela@msu.edu

**Keywords:** animal welfare volunteer satisfaction, perceptual surveys, common method bias, policy perception versus policy

## Abstract

**Simple Summary:**

There is a large body of work spanning decades examining the factors that lead to volunteer satisfaction. Much of this work has employed self-administered surveys to gather volunteers’ opinions or perceptions about factors important to satisfaction which inherently lie within the individual volunteer. Yet scholars have raised concerns about the validity of studies that rely on volunteers’ opinions or perceptions. This research pairs a survey of animal welfare volunteers with one conducted with the executive directors of the shelters they volunteer with. It concludes that perceptions appear to conform well to policy as reported by shelter directors but that volunteer perceptions are most important for satisfaction. The findings are critical to animal sheltering because they point to the importance of volunteer perceptions of shelter policies for volunteer satisfaction. To retain volunteers, animal welfare organizations need to be concerned about actual policies but also about volunteers’ perceptions of them.

**Abstract:**

There is a large body of work spanning decades examining the factors that lead to volunteer satisfaction. Much of this work has employed self-administered surveys to gather volunteers’ opinions or perceptions of factors important for satisfaction which inherently lie within the individual volunteer. Yet scholars have raised concerns about the validity of studies based on perceptions. This research builds on past work by the author by pairing a survey of animal welfare volunteers with one conducted with the executive directors of the shelters they volunteer with, addressing concerns about the validity of self-administered surveys. It concludes that perceptions appear to conform well to actual policies as described by shelter directors but that volunteer perceptions are most important for satisfaction. Volunteers whose perceptions of shelter policies differ from shelter director reports of policies are more likely to be dissatisfied with their experiences. The findings are critical to animal sheltering because they point to the importance of volunteer perceptions of shelter policies for satisfaction. To retain volunteers, animal welfare organizations need to be concerned about actual policies but also about volunteer perceptions of them.

## 1. Introduction

### 1.1. Animal Welfare Volunteering

Understanding what contributes to animal welfare volunteer satisfaction is important because of the nature of the work itself; it raises the caring-killing paradox whereby staff and volunteers often care for animals that they know the organization will euthanize, raising concerns over compassion fatigue [1] and creating a “moral stressor” which can exacerbate stress [2]. As with some other professions and volunteer settings, (nursing, public safety, domestic violence shelters) it involves “dirty work” with live subjects that can be physically risky and challenging (bites and disease, tasks requiring strength), and, depending on the activity, it necessitates special skill sets [3,4,5]. Animal shelter work has also been described as a “calling”, in that the activities involved are “morally, socially, and personally significant” to the individuals involved ([6], p. 584). While callings can be positive, they can also lead to stress, disillusionment, compassion fatigue, and costs to personal well-being [7,8]. And even though animal shelter volunteers may have a calling to do the work, there is a positive relationship between tenure and satisfaction, i.e., when they are dissatisfied, they quit [9]. 

There is a large body of work spanning decades examining the factors that lead to volunteer satisfaction (for systematic reviews of this literature see [10,11,12,13]). Particularly in animal welfare, volunteers are critical to achieving organizational missions. Attracting and retaining volunteers can be expensive and time consuming and turnover can cause disruption to the ability of shelters and rescue centers to provide necessary animal care and services to the community. 

### 1.2. Measuring Volunteer Perceptions

It is natural that research has focused on volunteer satisfaction and retention. Much of this work has employed self-administered surveys to gather perceptual data on factors important for satisfaction which inherently lie within the individual volunteer [14,15]. Yet scholars have raised concerns about studies that rely on volunteer opinions or perceptions. Limitations include the use of self-reported measures that might be invalid due to problems with recall and the potential for respondents to provide answers they think the researchers want or that are socially acceptable [14,16]. Common method bias has also been raised as a concern about studies that use self-reported surveys to measure both independent and dependent variables drawn from the same survey [17,18,19]. 

Suggested remedies for these concerns include surveying volunteers, staff, and clients to provide a more balanced and nuanced picture of satisfaction [14,20] or to pair volunteer surveys with organizational data, such as volunteer hours, tenure, or turnover [15,17,21]. Extant studies focused on organizational administrative data have had limited explanatory power because volunteer perceptions are not considered [21]. To address this requires research that not only includes volunteer and organizational perspectives but that is conducted across organizations [11]. Further, the effectiveness of organizational policies may well depend on volunteer perceptions of them [20], thus both factors should be examined in tandem [11,21]. It is expected that the use of volunteer surveys and organizational factors will improve the power of models seeking to account for volunteer satisfaction [21]. In other words, “people’s evaluation of objective working conditions (job satisfaction) may be only partially explained by the objective working conditions faced by workers” ([22], p. 936]) and different volunteers can perceive actual policies differently based on a variety of individual factors [20]. 

This research addresses these concerns by pairing a survey of animal welfare volunteers with one conducted with the executive directors of the shelters they volunteer with. The surveys were conducted at two points in time and non-survey-based measures of organizational attributes from secondary sources were also included, addressing calls to minimize bias in perceptual surveys [19]. The research addresses the following questions in the context of animal shelter volunteering:

1. Do volunteer perceptions accurately represent organizational policies?
2. Is volunteer satisfaction more strongly related to perceptions or policies?
3. Is the gap between perceptions and policy related to satisfaction? What types of volunteer are more likely to have lower perception/policy differentials?

## 2. Literature Review

### 2.1. Correlates of Volunteer Satisfaction

Extant research has identified a number of variables—volunteer demographics, motivations, training, volunteer voice in decision-making, policy transparency, volunteer/staff relationships, gratitude—that might impact satisfaction, although many of the findings have been mixed [23,24,25,26,27]. A meta-analysis of volunteer satisfaction in nonprofits based on 87 studies identified a number of critical determinants, including communication, training, clearly defined roles, recognition, and peer support [12]. A systematic review of 386 research publications on volunteer coordination indicated that organizational values and culture and attitudes towards volunteers were important to volunteer satisfaction [11]. 

Other work has suggested that the roots of volunteer satisfaction lie within the volunteers themselves. Research suggests that volunteer traits, such as gender, age, and education affect satisfaction with the amount of input volunteers have in their organization [28]. Some studies have found that older volunteers have greater satisfaction, experience less burn-out, have longer tenures, and contribute more hours per week than younger volunteers [24]. More educated volunteers report lower levels of satisfaction when their expectations are not met [12,29]. Six motives for volunteering have been identified: expression of altruism; learning new skills or gaining understanding; becoming part of a social network; gaining access to a job or career advancement; compensating for negative personal feelings; and enhancing personal self-esteem [30]. Research specifically on animal welfare volunteers has found only three motivations: careerism, altruism, and socio-emotional needs [27]. Volunteers motivated by altruism and socio-emotional factors appear more satisfied than those motivated by career enhancement [23,24,27,31]. Despite these findings, other research has suggested that organizational practices are more important for volunteer satisfaction than attributes or motivations of the volunteers [13]. 

### 2.2. Policies to Increase Volunteer Satisfaction

To increase volunteer satisfaction, it has been recommended that organizations incorporate opportunities for volunteers to share in planning and assessment of practices, including opportunities to provide feedback, often referred to as “voice” [32]. Volunteer satisfaction is enhanced by participation in meetings and volunteer representation on boards [27]. Negative interpersonal relationships with staff are one of the primary reasons volunteers leave an organization [33]. Development of social networks with other volunteers and between staff and volunteers is important for volunteer satisfaction and retention across a wide array of organizational types, including animal shelters and rescue centers [23,24]. However, other research has suggested that staff may be less than positive about volunteers for a variety of reasons, hindering these efforts: they fear that volunteers will take over their jobs; volunteers may be unfriendly; they may feel that volunteers do not know what they are doing and thus inhibit staff efforts to do their jobs; and, in some cases, they may feel intimidated by volunteers with more skills and experience working with animals or in shelters making them hesitant to more effectively make use of skills that volunteers developed in their outside careers [34]. The end result is that volunteers may be limited in the extent to which they can become more fully involved in shelters through activism. 

Providing more information to both volunteers and the public about an organization and its policies and processes also increases volunteer satisfaction [27,35]. Having clear and transparent policies appears important for volunteer satisfaction [32] and volunteers prefer more routinized activities with detailed instructions [36]. Training and role mastery appear to lead to increased efficacy and satisfaction among volunteers [29,37]. Finally, organizational expressions of gratitude (awards, dinners, or even just a thank you) appear to enhance satisfaction and intention to keep volunteering; these effects are more pronounced than for training [21]. In short, perceived organizational support via training and gratitude and feelings of personal importance within the organization through voice and positive relations with staff enhance satisfaction and commitment to the organization [31].

The current research is organized around the three questions previously presented since there is a dearth of extant studies comparing volunteer opinions and perceptions of shelter policies to the actual policies as reported by shelter directors. As a result, there is no basis on which to hypothesize about the extent to which perceptions match policies. However, the following hypotheses are posed regarding the second and third research questions:

**H_1_:**  *Volunteer satisfaction will have a stronger relationship with how individuals perceive shelter policies than the policies reported by shelter directors*.

**H_2_:**  *Larger differences between policies as perceived by volunteers and those reported by shelter directors will be related to lower volunteer satisfaction*.

## 3. Materials and Methods

The data for this study were drawn from two separate surveys conducted at different points in time; one of animal shelter directors, the other of their volunteers (i.e., both surveys were conducted at the same organizations). Quantitative data about the organizations, including live release rates, were collected from a secondary source. As a result, the methodology addresses central concerns about common method bias. All material for both surveys was reviewed and determined to be exempt by the Michigan State University Institutional Review Board for human subject research (IRB numbers 00002306 for the shelter director survey and x15-1091e; i049817 for the volunteer survey). 

### 3.1. Procedure: Volunteer Survey

All licensed animal shelters in the State of Michigan (*n* = 185 at the time of this survey in 2019) were invited to allow their volunteers to participate in the study via an email sent to the executive director. Fifteen agreed to distribute the survey. Directors were asked to forward a link to an online survey (hosted by Qualtrics) to all current volunteers. A total of 651 volunteers responded and 353 were matched with their shelter directors and were used in this study. A total of 63 questions constituted the volunteer satisfaction survey. The procedures for the development, piloting, and content of the survey questions and informed consent are discussed in more detail in prior work [27].

The respondents to the volunteer survey were overwhelming female (88%), married (51%), did not have children under 18 in the home (81%), and had a college degree or some college education (46% and 21%, respectively). They were relatively newer volunteers, working 1–3 years (35%) or less than a year (32%), and volunteered either 1–2 h (30%) or 2–4 h (28%) per week. The plurality of respondents were between 55 and 64 years (21%), followed by 25–34 and 35–44 years of age (both at 16%). 

### 3.2. Procedure: Shelter Director Survey

The population for this portion of the study was also all licensed shelters in the State of Michigan at the time of the survey in the fall of 2016: *n* = 165. A link to an online survey was sent to all executive directors, 93 of whom answered the survey, a response rate of 56%. Executive director responses were matched to the 15 shelters that distributed the volunteer survey; i.e., only 15 of the 93 responses were used to be able to match shelter director reports of policies to volunteer perceptions of them. The survey covered characteristics of the facility, programs conducted, volunteers, animal health care, adoption processes and fees, and assessment of goal attainment. The questions were based on best practice guidelines promulgated by the National Animal Care and Control Association (NACA), the Association of Shelter Veterinarians (ASV), the Society of Animal Welfare Administrators (SAWA), the Association for the Prevention of Cruelty to Animals (ASPCA), and the Humane Society of the United States (HSUS). Greater detail on the survey methodology can be found in previous research [38].

Characteristics of the organizations were drawn from yet another data source, the Michigan Department of Agriculture and Rural Development (MDARD) annual shelter reports; (1) save or live release rates and (2) annual intake of animals and intake method (stray, relinquishment, cruelty case, transfer). These data were drawn from 2018 to match the year of the more recent volunteer survey. Based on a comparison of responding shelter traits and all licensed shelters in the state reporting to the MDARD, all shelter types are represented: nonprofit/municipal; open/limited intake; animals served; and size. 

### 3.3. Analysis

First, factor analysis was used to reduce the number of variables to more parsimonious indexes and to reduce multicollinearity in the regressions to follow. Table 1 presents the results of factor analyses used to build the indexes used in the study. For the factor analysis, the principal components method with varimax rotation was employed. 

The motivations of the volunteers loaded on three separate factors indicating career motivations, socio-emotional motivations, and altruism motivations. Attributes of the animal shelters are included in a single index representing potential sources of *shelter stress*: higher euthanasia rates, larger intakes, and higher stray intakes. Shelter programs are categorized by the types of *dog enrichment* provided, the comprehensiveness of *medical care* for the animals, opportunities for volunteers to have voice or a say in decision-making (*shelter voice*), the extent to which the organization is transparent to volunteers (*shelter transparency*), and the extent of training provided to volunteers (*shelter volunteer training*). There are four indexes measuring volunteer *perceptions* of shelter policies regarding training (*perceived training*), gratitude (*perceived gratitude*), voice or a say in decision-making (*perceived voice*), transparency (*perceived transparency*), and how friendly volunteers find staff and their volunteer colleagues (*perceived friendliness*). Finally, four questions comprise the *volunteer satisfaction index*. All of the volunteer perceptional and shelter policy questions have been employed in previous research using data from surveys of volunteers and shelter directors combined into similar indexes [9,13,27]. 

Bivariate correlation analysis is employed to explore the significant relationships between the volunteer satisfaction index and the variables representing volunteer demographics and motivations; shelter policies as reported by directors; the variables measuring shelter stress; and policies as perceived by volunteers. This is followed by a series of OLS regression analyses. Four different regressions are run to compare the explanatory power of each set of independent variables: volunteer traits and motivations, shelter stress, shelter policies as reported by directors, and policies as perceived by volunteers. A fifth and final regression represents the best fitting model including only those variables significantly correlated with satisfaction in the preceding regressions.

## 4. Results

### 4.1. Bivariate Correlations

The analysis begins by identifying the significant correlates of volunteer satisfaction (Table 2). None of the volunteer demographics are significantly correlated with satisfaction, however, motivations for volunteering are. Respondents higher on all of the motivation indexes are significantly more satisfied with their experience. Volunteers that *perceive* that they have input in organizational decision-making (voice), that their training is sufficient, and that their shelter is friendly and transparent are significantly more likely to be satisfied with their experience. Volunteers in shelters that provide more avenues for voice, which are more transparent, have more activities and practices that provide enrichment to shelter dogs, and that provide more medical care are significantly more satisfied. Finally, several attributes of the shelters themselves are significantly correlated with satisfaction. Volunteers indicate that they are more satisfied if they are with shelters that have higher live release rates, lower yearly intake of animals, and have fewer stray intakes. This conforms to prior research finding that volunteers in shelters with better outcomes for animals tend to be more satisfied [38]. In short, perceptions of policy, policies as reported by shelter directors, volunteer motivations, and several attributes of the shelter are significantly correlated with volunteer satisfaction. The regression analyses to follow in Section 4.2 explore the relative explanatory power of these variables. 

Table 3 presents the correlations between volunteer perceptions of shelter policy and actual organizational policies as reported by shelter directors with the goal of answering the first research question, do volunteer perceptions accurately represent organizational policies? The two surveys did not include all of the policy questions so that only those questions that were included on both surveys are employed in this analysis: voice index, training index, and transparency index. Volunteer perceptions of the extent of their voice in the shelter are significantly correlated with director reports of policies to ensure voice. Volunteer perceptions of the extent of their training are significantly correlated with director reports of training used in the shelter. Finally, volunteer perceptions of the extent of transparency in shelter activities are significantly correlated with director reports of efforts to be transparent to volunteers. 

### 4.2. Regression Analyses

To answer the third research question, the regression analyses allow for identification of what types of variables best explain satisfaction. Again, four different regressions are presented to compare the explanatory power of each set of independent variables: volunteer traits and motivations, characteristics of the shelter, shelter policies as reported by directors, and policies as perceived by volunteers. The last regression model represents the best fitting model including only those variables significantly correlated with satisfaction in the preceding regressions.

Table 4 presents the results of volunteer demographics and motivations regressed on volunteer satisfaction. This set of variables, representing factors internal to the volunteers, accounts for 24% of the variation in satisfaction. Only one variable remains significantly correlated to satisfaction in the model, however. Volunteers higher on the emotional motivation index are significantly more satisfied with their volunteer experience.

Table 5 presents the regression results for the shelter stress index regressed on volunteer satisfaction. This index accounts for only 3% of the variation in volunteer satisfaction, although the relationship between shelter stress is significantly and negatively related to volunteer satisfaction. Volunteers in shelters with higher euthanasia rates, annual intakes, and annual stray intakes are significantly less satisfied, although these variables alone explain little of the variance in satisfaction. 

Actual shelter programs as reported by shelter directors account for only 6% of the variation in volunteer satisfaction (Table 6). Volunteers at organizations that provide more opportunities for voice and input are significantly more satisfied with their volunteer experiences. The extent of shelter transparency as reported by directors does not remain significantly correlated to satisfaction in multiple regression, nor does the extent of enrichment provided to dogs. 

Table 7 provides the results of the regression model with satisfaction as the dependent variable and volunteer perceptions of shelter policies as the independent variables. This model has the strongest explanatory power, accounting for 79% of the variance in satisfaction. Volunteer perceptions are more important for satisfaction than reported shelter policies, traits of the shelter, or attributes of the volunteers. Volunteers are more satisfied if they perceive that their shelter provides more training, is more transparent, offers more opportunities for voice in decision-making, and provides a friendly environment. The perception of friendliness followed by perceptions of voice is the strongest predictor of satisfaction based on the beta values.

Table 8 provides the results of the full, best fitting, regression model including only those independent variables that were significantly correlated to satisfaction in the preceding analyses. This reduced full model accounts for 80% of the variance in volunteer satisfaction. Volunteers are more satisfied if they perceive that their shelter provides more opportunities for training, voice, and is friendly. Actual opportunities for voice as reported by shelter directors do not remain significantly correlated with satisfaction in the model. Stress on the shelter in terms of euthanasia and higher annual intakes also does not remain significantly correlated with volunteer satisfaction in the model. Finally, volunteers that are driven by socio-emotional motivations are significantly more satisfied. Of the variables in the model, perception of friendliness is the best predictor of satisfaction based on the beta values. It should be noted that transparency as perceived by the volunteers was left out of this regression model due to multicollinearity with the friendliness index. 

### 4.3. Differences between Perceptions and Shelter Policy

The preceding regression analyses address the second research question. To answer the third and final research question (is the gap between perceptions and policy related to satisfaction?), differential variables were calculated by subtracting volunteer perception index scores from the policy index scores as reported by shelter directors. In all cases, a lower (negative) value indicates that a volunteer’s perceptions are closer to policy as reported by directors. Volunteers whose perceptions more closely match actual organizational policies are significantly more satisfied with their volunteer experience (Table 9). 

Table 9 also presents correlations between traits of the volunteer, differences between volunteer perceptions of policy and policy as reported by directors, and volunteer satisfaction. Demographic variables are included to address the second part of the last research question regarding which volunteers are more likely to have perceptions closer to the policies as reported by directors. 

The volunteers with lower perceptual/policy differentials, meaning that their perceptions more accurately reflect policy as reported by directors, are those that are motivated by altruism and socio-emotional needs. Those higher on career motivations have lower differentials for voice and training but not for transparency. In other words, career motivated volunteers’ perception of the extent of shelter transparency is more likely to deviate from actual policy. Respondents that have longer tenures with their organizations have perceptions closer to reported policies for voice and training, but have a significantly higher differential for transparency, i.e., more experienced volunteers appear to perceive transparency differently than shelter director reports of transparency. Similarly, those volunteering more hours per week have lower differentials for training but higher differentials for transparency. Finally, education appears to widen the gap between perceptions and policy for training and transparency, with more educated volunteers less likely to have perceptions that match shelter director reports, conforming to previous research indicating that more education may cause volunteers to question policies [13,34].

## 5. Discussion

Three questions were posed in this research (1) Do volunteer perceptions accurately represent organizational policies? (2) Is volunteer satisfaction more strongly related to perceptions or policies? (3) Is the gap between perceptions and policies as described by shelter directors related to satisfaction?; What types of volunteers are more likely to have lower perception/policy differentials? Regarding these questions, two hypotheses were also posed:

**H_1_:**  *Volunteer satisfaction will have a stronger relationship with how individuals perceive shelter policies than the policies reported by shelter directors*.

**H_2_:**  *Larger differences between policies as perceived by volunteers and those reported by shelter directors will be related to lower volunteer satisfaction*.

First, the results indicate that animal welfare volunteers are accurate in their perceptions of organizational policies as reported by shelter directors (Table 3). For practice, this means that shelter administrators can generally be confident that volunteers can accurately report on shelter policies and are aware of efforts to include them in decision-making, opportunities for social interaction, and training opportunities. And, since volunteer perceptions of these policies are correlated with satisfaction, shelter professionals can likely increase volunteer satisfaction and retention by emphasizing such policies (Table 2 and Table 7), as suggested in previous work [13,26,27]. From a research methodology perspective, the findings suggest that surveys of the perceptions of volunteers accurately reflect policies and procedures of the organization. Thus, concerns about the validity of self-reported measures may be overstated in previous work, at least in the context of animal welfare volunteers [14,15,16].

Second, comparing across the separate regression analyses (Table 4, Table 5, Table 6 and Table 7) indicates that volunteer perceptions account for more of the variation in satisfaction than actual policies as reported by shelter directors, traits of the organization such as intake and euthanasia, or the demographic characteristics and motivations of the volunteers themselves, supporting H_1_. However, looking within the individual regressions it also appears that socio-emotional needs fulfillment, social interaction, and feelings of importance within the organization are critical to volunteer satisfaction and commitment (Table 8), in line with previous work [13,26,27]. These findings suggest that while volunteers as a whole correctly perceive policies that are in place, there is still room for shelter administrators to engage in efforts to clearly inform volunteers about shelter policies and opportunities for voice, training, and social interaction. The final reduced model also suggests that volunteers higher on socio-emotional motivations—volunteering makes them feel better, happier with themselves, and less lonely—are more satisfied (Table 8). Shelter directors and volunteer coordinators may want to explore the motivations of perspective volunteers to identify those that may be predisposed to find animal welfare volunteering more personally fulfilling. Volunteer recruitment and orientation efforts could include discussions of motivations. 

Third, smaller differences between perceptions of shelter policies and reported policies increase satisfaction, supporting H_2_ (Table 9). Thus, as suggested in some previous research, it is how volunteers perceive policies that is critical to satisfaction [20]. More specifically, while greater involvement with the shelter generally brings perceptions and policy closer together, it appears that it may make volunteers more skeptical about transparency. This implies that more experienced volunteers that have had greater interaction with shelter policies may have a tendency to feel that the organization is not being as clear as possible about internal processes and procedures. Shelter directors and volunteer coordinators may want to consider different communication modalities and messages targeted at volunteers with different experience and tenure levels [26].

## 6. Conclusions

First, it appears that self-administrated surveys of volunteer perceptions provide valid indicators of organizational policies. Second, even without a perfect correlation between perceptions and reported policy, it is volunteer perceptions that matter most to satisfaction. How volunteers feel about the amount of input they have in decision-making and how friendly they perceive staff and other volunteers to be are the most important correlates of satisfaction. This supports other research emphasizing the importance of perceived organizational support for volunteer satisfaction and retention [14,15]. Based on the findings here, this appears to be particularly important for more senior volunteers. Making affirmative efforts to be transparent about shelter policies is important to all volunteers, but may be particularly critical to those that have been with the organization for a longer period of time. Ensuring the retention of these most experienced volunteers is worth targeted information provision. Additionally, animal welfare organizations should not only strive to maintain a friendly and welcoming environment and allow volunteers to have a voice in policymaking, but they also need to ensure that volunteers are aware of these efforts. Regular internal surveys of volunteers would allow organizations to assess perceptions of friendliness and voice. Providing opportunities for staff and volunteers to interact with each other should increase perceptions of friendliness but also break down some of the barriers between staff and volunteers noted in previous research [34]. The findings are important to animal sheltering because they point to the impact of volunteer perceptions of shelter policies on satisfaction and likely retention based on research findings that more satisfied volunteers have longer tenures [13]. To retain volunteers, animal welfare organizations need to be concerned about actual policies but also volunteer perceptions of them. 

Finally, this research makes a significant contribution to methodology surrounding the use of self-administered perceptional volunteer surveys to measure the nature of policies and procedures used by the organizations they volunteer with. Prior research using data from the shelter director questionnaire explored the internal shelter policies related to better outcomes for the animals in terms of higher live release rates [38], but attitudes among the volunteers about those policies were not assessed. Data from the volunteer survey were employed in earlier work to assess which policies were most strongly related to satisfaction [13,26,27]. However, these studies did not examine whether volunteer perceptions of shelter policies actually matched the extant policies in place. The current study is the first to combine volunteer and shelter director data in the same analysis, allowing for a unique comparison of perceptions and policy within the same shelters. The significant correlations between volunteer perceptions and actual policies supports the validity of methodologies that employ volunteer surveys as a proxy for organizational policies. 

## 7. Limitations

This research has some limitations. First, it relies on data from a small number of organizations; larger samples with volunteers across different countries may yield different results. Second, because the project used data from surveys at two points in time and organizations could have changed their policies in the interim, it could be that volunteer perceptions reflect different policies. This potential for error was not possible to address in this research, but future work should employ staff and volunteer companion surveys conducted at the same point in time. Third, the two surveys were designed for different purposes and research projects. Although the data represent volunteers matched to their organizations, the survey questions used to measure the concepts of voice, training, and transparency differed somewhat. While the strong correlation between perceptions and policy as reported by shelter directors suggests that this was not problematic, it could be a cause of measurement error. Finally, just as volunteer perceptions may be biased, policies may be misrepresented by organizational leaders. 

## Figures and Tables

**Table 1:** Factor Analysis.

	Factor Loading
Shelter Stress	
Euthanasia rate	0.995
Stray intake	0.62
Total intake	0.87
Dog enrichment	
Behavior plans	0.81
Pack hikes	0.73
Play dates	0.87
Agility	0.84
Day trips	0.89
Foster care	0.90
Enrichment toys/activities	0.86
Outdoor play yard	0.69
Indoor play area	0.66
Reading program	0.73
Running program	0.61
Chew toys	0.86
Medical care	
Document all care	0.98
Assess health on intake	0.98
Core vaccines	0.98
Modified live vaccines	0.74
Rabies	0.98
Emergency medical plan	0.98
Pain management	0.80
Parasite treatment/preventatives	0.98
Groom	0.94
Disease response plan	0.98
Spay tattoo	0.80
Shelter Transparency	
Organization chart	0.83
Community booth	0.77
Kids programs	0.87
Tours	0.91
Day of service	0.87
Shelter Volunteer Training	
Apprenticeships	0.88
Training for specific tasks	0.84
Training updates	90
Shelter Voice	
Volunteer board	0.80
Volunteer surveys	0.80
Volunteer Satisfaction	
Satisfied with overall experience	0.84
Shelter makes good use of talents/skills	0.84
Volunteers treated with respect	0.85
Do not think of quitting	0.82
Perceived Transparency	
Transparent about animal data	0.79
Transparent about volunteer data	0.78
Shelter helps other shelters in area	0.77
Shelter transports animals from other areas	0.78
Perceived Gratitude	
Receive formal recognition	0.90
Shelters should recognize volunteers	0.40
Recognition makes me feel appreciated	0.86
Perceived Friendliness	
Other volunteers friendly	0.80
Staff friendly	0.86
Made friends with volunteers	0.60
Everyone is a team	0.86
Opportunities to interact with staff	0.85
Staff are helpful	0.84
Perceived Training	
Had apprenticeship	0.81
Hands-on experiences	0.85
Training updates	0.75
Had enough training	0.51
Perceived Voice	
I am able to have input in shelter policies and practices	0.79
I agree with policies and procedures	0.83
There are no negative consequences when I make suggestions	0.77
Career Motivations	
Get foot in the door	0.87
Looks good on resume	0.87
Socio-Emotional Motivations	
Helps me feel better if feeling bad	0.79
To feel less lonely	0.81
Feel better about myself, fulfilled	0.79
Altruism Motivations	
Concerned about homeless animals	0.85
I can make a difference	0.85

**Table 2:** Pearson Correlations Volunteer Satisfaction.

	Volunteer Satisfaction
Demographic Variables	
Gender ^ (male)	10.49
Age	−0.05
Education	0.01
Marital status ^	2.70
Children under 18 ^	2.50
Volunteer Perceptions	
Training	0.54 **
Friendly	0.84 **
Gratitude	0.46 **
Transparency	0.61 **
Voice	0.82 **
Motives for volunteering	
Careerism	0.30 **
Emotional	0.48 **
Altruism	0.32 **
Shelter Policies	
Shelter voice	0.26 **
Shelter training	0.08
Shelter transparency	0.52 *
Dog enrichment	0.18 **
Medical care	0.14 *
Attributes of Shelter	
Save rate	0.23 **
Annual intake	−0.13 *
Stray intake	−0.17 **
Relinquishment intake	0.12
Cruelty/rescue intake	−0.04
Transfer intake	0.22

^ Chi square * sig at 0.05 ** sig at 0.01.

**Table 3:** Pearson Correlations, Volunteer Perceptions and Shelter Policy.

	Shelter Voice	Shelter Training	Shelter Transparency
Volunteer voice	0.26 **	0.09	0.22 **
Volunteer training	0.33 **	0.32 **	0.30 **
Volunteer transparency	0.17 **	0.10	0.13 *

* sig at 0.05 ** sig at 0.01.

**Table 4:** Regression, Volunteer Satisfaction and Volunteer Traits.

	Unstandardized b	Standard Error	Beta	t	sig	VIF
Gender	0.10	0.06	0.10	1.61	0.11	1.06
Age	−0.02	0.04	−0.03	−0.46	0.64	1.12
Education	0.02	0.05	0.03	0.46	0.65	1.07
Marital status	0.00	0.06	0.00	−0.01	0.99	1.04
Children under 18	0.27	0.15	0.11	1.79	0.07	1.06
Careerism	0.12	0.07	0.12	1.80	0.07	1.24
Emotional	0.42	0.07	0.42	6.02	0.00	1.41
Altruistic	0.09	0.06	0.09	1.39	0.17	1.30
Constant	-0.63	0.38		−1.63	0.10	
Adjusted R^2^ = 0.24						

**Table 5:** Regression, Volunteer Satisfaction and Nature of Organization.

	Unstandardized b	Standard Error	Beta	t	sig
Shelter stress	−0.18	0.07	−0.18	−2.79	0.00
Constant	−0.09	0.06		−1.41	0.16
Adjusted R^2^ = 0.03					

**Table 6:** Regression, Volunteer Satisfaction and Shelter Programs Reported by Directors.

	Unstandardized b	Standard Error	Beta	t	sig	VIF
Shelter transparency	0.11	0.13	0.11	0.87	0.39	4.14
Dog enrichment	0.12	0.13	0.11	0.91	0.37	4.07
Shelter voice	0.26	0.08	0.26	3.20	0.00	1.75
Constant	−0.09	0.06		−1.38	0.17	
Adjusted R^2^ = 0.06						

**Table 7:** Regression, Volunteer Satisfaction and Volunteer Perceptions of Programs.

	Unstandardized b	Standard Error	Beta	t	sig	VIF
Training	0.09	0.04	0.09	2.26	0.03	1.50
Friendly	0.44	0.05	0.44	9.45	0.00	2.28
Transparency	0.09	0.04	0.09	2.20	0.03	1.66
Voice	0.42	0.05	0.40	8.12	0.00	2.70
Constant	0.00	0.03		−0.01	1.00	
Adjusted R^2^ = 0.79						

**Table 8:** Regression, Volunteer Satisfaction, Full Model, Reduced.

	Unstandardized b	Standard Error	Beta	t	sig	VIF
Shelter stress	−0.03	0.04	−0.03	−0.72	0.47	1.55
Shelter voice	0.03	0.04	0.03	0.85	0.39	1.51
Training	0.08	0.04	0.07	1.84	0.07	1.64
Friendly	0.44	0.05	0.43	9.24	0.00	2.29
Volunteer voice	0.43	0.05	0.42	8.80	0.00	2.38
Emotional motives	0.14	0.04	0.14	3.96	0.00	1.29
	−0.02	0.03		−0.54	0.59	
Adjusted R^2^ = 0.80						

**Table 9:** Pearson Correlations Perception/Policy Differentials, Satisfaction, and Volunteer Traits.

	Voice Differential	Training Differential	Transparency Differential
Satisfaction	−0.35 **	−0.67 **	−0.31 **
Careerism	−0.23 **	−0.22 **	0.04
Emotional	−0.31 **	−0.25 **	−0.20 **
Altruism	−0.20 **	−0.17 **	−0.15 *
Gender ^	12.65	8.92	9.25
Hours per week	0.02	−0.15 *	0.18 *
Tenure	−0.26 **	−0.33 **	0.18 **
Age	−0.08	−0.04	−0.04
Education	0.05	0.16 *	0.20 **
Marital status ^	3.78	12.29	7.23
Children under 18 ^	0.00	4.80	0.17

^ Chi square * sig at 0.05 ** sig at 0.01.

## Data Availability

Data are available in the manuscript.
